# Structuring privacy policy: an AI approach

**DOI:** 10.3389/frai.2025.1720547

**Published:** 2026-01-14

**Authors:** Shani Alkoby, Ron S. Hirschprung

**Affiliations:** Faculty of Engineering, Industrial Engineering and Management, Ariel University, Ariel, Israel

**Keywords:** artificial intelligence, human computer interaction, machine learning, policy, privacy

## Abstract

**Introduction:**

Privacy has become a significant concern in the digital world, especially concerning the personal data collected by websites and other service providers on the World Wide Web network. One of the significant approaches to enable the individual to control privacy is the privacy policy document, which contains vital information on this matter. Publishing a privacy policy is required by regulation in most Western countries. However, the privacy policy document is a natural free text-based object, usually phrased in a legal language, and rapidly changes, making it consequently relatively hard to understand and almost always neglected by humans.

**Methods:**

This research proposes a novel methodology to receive an unstructured privacy policy text and automatically structure it into predefined parameters. The methodology is based on a two-layer artificial intelligence (AI) process.

**Results:**

In an empirical study that included 49 actual privacy policies from different websites, we demonstrated an average F1-score > 0.8 where five of six parameters achieved a very high classification accuracy.

**Discussion:**

This methodology can serve both humans and AI agents by addressing issues such as cognitive burden, non-standard formalizations, cognitive laziness, and the dynamics of the document across a timeline, which deters the use of the privacy policy as a resource. The study addresses a critical gap between the present regulations, aiming at enhancing privacy, and the abilities of humans to benefit from the mandatory published privacy policy.

## 1 Introduction

In the current digital era, privacy concerns have become an issue of great importance (Nippert-Eng, 2019), enveloping us in almost every online interaction. For example, engaging in e-commerce transactions poses the risk of exposing personal sensitive data (Muneer et al., 2018), which may include users’ commodities preferences (Wu et al., 2021); The utilization of e-health technology generates medical records that could potentially fall into the wrong hands, severely compromising our privacy (Chenthara et al., 2019); Online social networks are often seen as tools that intrude upon and undermine privacy (Van Schaik et al., 2018); The adoption of artificial intelligence (AI) to accelerate scientific processes is sometimes based on sensitive personal data that may leak (Lawrence and Montgomery, 2024); and many location based applications introduce a significant privacy concern (Zakhary and Benslimane, 2018). Search engines unveil information about our interests (Liu and Li, 2018); The emerging Internet of Things (IoT) technology has the potential to reveal details about our daily activities (Sharma et al., 2020); and even the encryption of domain names, which is usually overlooked, provides insufficient protection of our privacy while browsing the internet (Hoang et al., 2021). Collectively, these examples underscore the crucial nature of the privacy issue, positioning it as a central and vital concern (Jin et al., 2019; Mouloua et al., 2019; Paul and Aithal, 2019; Lutz et al., 2018).

As of today, privacy is recognized as a universal concern. This reality is significantly amplified by the presence of AI tools, which are perceived as threats to privacy (Elliott and Soifer, 2022). Western nations acknowledge privacy as an essential human right (Regan, 2002), actively pursuing regulations aimed at preserving privacy (Mokrosinska, 2018). Among these nations, the European Union is considered a leader, largely due to initiatives like the General Data Protection Regulation (GDPR), which seeks to enhance privacy protections for individuals (Dorraji and Barcys, 2014; Li et al., 2019). Similarly, in many Western jurisdictions, laws such as the California Business and Professions Code’s Internet Privacy Requirements (CalOPPA) mandate that commercial websites and online services that collect personally identifiable information must adhere to specific provisions outlined in their privacy policies (California Law, 2003). As a result, data protection policies are enforced on websites (Gopal et al., 2023).

While an effective policy may exist (due to regulation), a key tool in protecting privacy is the privacy notice, which informs individuals about how their sensitive data is handled. It should detail if and why personal data is collected (purpose), how it is used (method), how long the data is retained (retention), and relevant legal factors (entity and legality) (NHS 75, 2023). Privacy decision-making by an individual in the digital era is complicated (Bélanger and James, 2020), therefore, the concept of the ‘privacy pragmatist’, which suggests that users make rational, informed decisions about their data, has dominated privacy discussions, despite evidence challenging this model (Draper, 2017). Regardless of the difficulties, a necessary condition to be able to make these decisions is that the privacy terms are available to this user. The GDPR particularly regulates privacy notices, emphasizing mandatory transparency (Feth, 2017). In the digital world, privacy notices are frequently referred to as privacy policies. Additionally, the ‘notice’ often directs readers to the privacy policy document. These policies articulate in natural language how organizations or agencies manage individuals’ personal information (AG, 2023). Most websites, including major ones like Google search engine (Google, 2023), the online social network, Facebook (Meta, 2023), the e-commerce site AliExpress (Alibaba Group, 2022), and the US government’s official webpage (USAgov, 2023), feature privacy policies.

Privacy policies are supposed to play a crucial role in the task of protecting privacy, as they furnish users with essential details regarding the utilization of their personal data, often serving as their primary, and even only, source of such information (Jensen and Potts, 2004). With the advent of GDPR, privacy policies now encompass a wider range of data practices, potentially leading to enhanced transparency in organizations (Linden et al., 2019). The information that is provided in the privacy policy refers not only to straightforward data that the user enters, e.g., a shopping list but also to indirect information. For example, many platforms provide location-based services (LBSs), which may create a significant privacy issue (Qing et al., 2024). Beyond meeting user expectations, privacy policies also play a crucial role in promoting privacy-conscious website development efforts (Earp et al., 2005).

While the privacy policy is vital, its utilization poses challenges for several reasons. First, the privacy policy document is designated for the average user, yet it inherently contains technical and legal aspects, resulting in *low readability*—making comprehension difficult even for the common user (Krumay and Klar, 2020). Second, beyond readability, the privacy policy is a *complex document*, objectively challenging for layman users to understand, let alone comprehend the potential consequences of actions taken, or not taken, regarding their privacy disclosure (Winkler and Zeadally, 2016). In practice, textual privacy policies in their current forms also make it challenging to comply with the GDPR. Therefore, some research proposed tools to convert privacy policies into software to achieve accountability (Rhahla et al., 2021). Third, the privacy policy is not static but dynamic, *subject to frequent changes* (Amos et al., 2021). These changes may be significant, requiring users to bear the significant burden of staying updated. For example, the evolving discourse around children’s privacy on platforms like TikTok highlights the relationship between public pressure and privacy policy updates, reflecting the dynamic nature of platform governance (Ingber and Su, 2024). Often, users are not notified, necessitating periodic sampling of the privacy policy. Furthermore, users tend to exhibit *cognitive laziness* (Puaschunder, 2021). Even if the privacy policy is readable, the task of reading it remains lengthy, boring, and exhausting. In practice, reading the privacy policy carries a *cost*, which may not be justified when considering the cost of privacy disclosure (McDonald and Cranor, 2008). Lastly, users often believe that privacy breaches will not personally affect them, fostering cognitive laziness and other factors that deter them from reading the privacy policy (Hinds et al., 2020).

The Facebook-Cambridge Analytica incident revealed the use of Facebook users’ data for training an algorithm aimed at influencing US presidential elections (Kanakia et al., 2019). However, it was noted that Facebook’s actions during that period did not substantially violate its privacy policy. Consequently, it is plausible that some of the 87 million users whose personal data were compromised could have avoided such exposure by familiarizing themselves with Facebook’s privacy policy. This incident indirectly underscores the challenges associated with the usability of privacy policies. Studies suggest that when privacy policies are not presented by default (opt-out), such as in the case of many websites, a significant proportion of users tend to disregard them (Steinfeld, 2016). To examine users’ attitudes toward privacy policy documents, we conducted a preliminary survey.

All participants of this survey gave their consent prior to participation. The research design and details were submitted and approved by the institutional ethics committee (approval number AU-ENG-RH-20230718 dated Jul. 2023). The anonymity of the participants was carefully preserved. The sample size included *n* = 103 valid participants. Findings revealed that 58% of respondents admitted to never, rarely, or only sometimes reading the privacy policy document, while only 19% claimed to read it most of the time, and 22% stated that they read it all the time. When asked to provide a definition of a privacy policy, only 40% of respondents responded accurately. Furthermore, when participants were questioned about their general privacy concerns, no statistically significant correlation was found between their level of concern and their engagement with privacy policy documents. A similar survey was administered to students enrolled in their last 2 years in a Bachelor of Science in Engineering program, who are expected to have high technological literacy. In response to the binary question, “Who has ever read the privacy policy?” only four of approximately 80 students indicated they had read it. Therefore, while the privacy policy has the potential to provide privacy protection in theory, it usually fails to do so.

An earnest effort to address this issue was undertaken by the Platform for Privacy Preferences Project (P3P), introduced by the World Wide Web Consortium (W3C, 2018). The concept behind P3P entailed the following steps. First, websites would declare their data collection practices in a structured format and in an easily accessible location, similar to a privacy policy. Next, users would install a browser agent and configure their privacy preferences. Finally, upon browsing a site, the agent would retrieve its privacy policy, compare it to the user’s preferences, and provide a notification indicating whether the site aligned with these preferences. The fundamental objective of this approach is to solve the privacy policy usability problem by presenting the privacy policy in a machine-readable format, thereby alleviating the burden of manual reading among users (Cranor, 2003). While P3P represented a commendable and innovative idea, its viability was limited, primarily by the requirement of cooperation from website owners and its perception as a complex solution (Schwartz, 2009; EPIC, 2000). Moreover, as P3P was a recommendation rather than a mandatory regulation, websites were not obliged to implement it. Given the voluntary nature of P3P adoption and the potential conflict of interest between organizational objectives and individual privacy concerns, such as employee email monitoring (Smith and Tabak, 2009), P3P faced significant challenges. A study conducted in 2007 revealed that only 8.3% of the e-commerce websites (of the 5,553 scanned) had adopted P3P (Beatty et al., 2007). Over time, P3P implementation has declined, is currently no longer supported by browsers and is rarely discussed.

This research is motivated by the shortcomings of the P3P initiative and enduring usability challenges associated with privacy policies. Our objective is to develop a robust machine-executable methodology for interpreting privacy policy documents. One that enhances accessibility for both human users and AI agents. To achieve this objective, the proposed solution must satisfy two essential requirements. First, human intervention should be substantially reduced or eliminated following the machine training phase. Second, the methodology must operate independently of any cooperation from website owners. Meeting these requirements allows us to address the barriers that currently hinder the effective use of privacy policies. Accordingly, the central research question is: How can privacy policies, which are inherently complex and typically expressed as legally oriented, unstructured free text, be transformed into a structured format that improves accessibility for users and AI-based automated assistants? Notably, AI systems often contribute to privacy risks by processing vast quantities of sensitive information, thereby increasing vulnerability to breaches (Hosseini and Seilani, 2025). This study therefore employs AI as a means of mitigating harm generated by AI itself.

The key contributions of this methodology are as follows: (a) It requires no cooperation from the provider, such as the website owner, who may not even be aware of the process. This design avoid potential resistance stemming from conflicts of interest; (b) The solution is fully automated, thereby overcoming barriers associated with users’ cognitive burden and inaction; (c) The privacy policy does not need to be reformatted or transformed such as into a unified template. This feature enables application to any policy that meets minimal regulatory requirements; (d) The proposition-based approach provides a flexible mechanism that can be adapted to future regulatory or structural changes; (e) This approach also allows the introduction of additional parameters for privacy-related classification; and (f) The empirical study is grounded in privacy policies from widely used contemporary websites, rather than a specialized or potentially unrepresentative corpus.

## 2 Literature review

The literature extensively addresses various aspects of privacy policy documents, with a plethora of sources available. There appears to be a consensus that, fundamentally, privacy policies lack readability. One possible definition of readability is “the degree to which a given class of people find certain reading matter compelling and comprehensible” (Mc Laughlin, 1969). While this early definition is pertinent to our context, the element of compulsion is not relevant to a non-obligatory document. Readability also encompasses User Interface (UI) elements such as font size, but these are less significant in digital non-printed media, because users can adjust them. Therefore, the focus is primarily on the content itself. Srinath et al. (2020) identified the existing challenges associated with natural language privacy policies and highlighted the potential for leveraging AI technologies such as natural language processing (NLP) to interpret these documents. However, they noted an absence of large-scale datasets suitable for training machine-learning (ML) models. To address this gap, they developed a corpus comprising over 1 million English-language website privacy policies, named ‘PrivaSeer’, and conducted readability assessments. Their findings revealed an average readability score of 40 (with a range of 0–100, where 80–100 indicates very easy to read and 0–20 indicates very difficult to read), indicating that privacy policies are generally challenging to comprehend. Robillard et al. (2019) demonstrated that the readability of privacy policies or terms of agreement (ToA) for mental health apps are “too difficult for the general population.” Discrepancies between user expectations and privacy policy practices in social media, particularly regarding informed consent, underscore the need for user-centric approaches to privacy policy design (Custers et al., 2014). It has also been demonstrated that the interaction type affects users’ attention regarding parts of the privacy policy—directly impacting the consent provided (Karegar et al., 2020). Jha et al. (2022) showed that the effect of accepting privacy policies (in practice consent), e.g., web tracking is far more pervasive, and web pages are larger and slower to load. Alić (2023) investigated the transparency aspect of privacy policies, emphasizing the importance of the language component in achieving this trait.

Efforts have been undertaken to standardize privacy policies using various languages, such as the Enterprise Privacy Authorization Language (EPAL) and the OASIS Standard eXtensible Access Control Markup Language (XACML) (Anderson, 2006). However, this approach bears resemblance to the P3P project and thus is susceptible to similar shortcomings, primarily complexity and a lack of cooperation. Kumaraguru et al. (2007) conducted a survey of privacy policy languages, including SAML, XACML P3P, CPExchange, PRML, and Geo-Priv, focusing on language structure. However, as previously mentioned, language structure is not the central issue, and while these solutions appeared promising at the time (around 2007), none achieved a significant breakthrough. Another similar approach is to pre-design the policy in a structural format to address specific requirements, e.g., security policies for NoSQL document databases (Blanco et al., 2022). However, this approach also requires the cooperation of the provider. Huang et al. (2019) proposed leveraging privacy-preserving techniques in recommendation systems to develop privacy policies aimed at safeguarding users’ privacy against breaches resulting from information leaks. This approach, rooted in the current method of accessing privacy policies, endeavors to enhance document readability by employing the concept of contextual integrity. This involves identifying missing contextual details, addressing vague language, and avoiding potential misinterpretations (Shvartzshnaider et al., 2019). While this approach may alleviate the burden on users, it necessitates cooperation on the part of the websites and may not be effective unless enforced by regulators.

A cutting-edge approach introduced a programming language called Jeeves to represent and enforce privacy policies (Yang et al., 2012). However, in addition to requiring cooperation on the part of the website owners, this solution is highly complex and practically infeasible to implement. Alternatively, Ghazinour and Albalawi (2016) proposed a radically different approach to address this issue through the visualization of privacy policies. While this method may alleviate user burden and enhance usability, it still relies on cooperation from websites. Furthermore, it may also raise legal concerns in this traditionally conservative field, as it is uncertain whether stakeholders will abandon written policies, potentially leading to dual representation—both textual and visual. Significant issues are anticipated regarding the compatibility of these two representations.

A significant step toward a viable solution was taken by integrating ML into this endeavor, effectively addressing the human burden and the need for cooperation (Costante et al., 2012), when this work may serve as a proof of concept and lay the groundwork for a satisfactory resolution. Sadeh et al. (2013) introduced an approach to this problem that combined ML and NLP with crowdsourcing. While their solution is semi-automatic and requires substantial efforts to implement, it represents a breakthrough in a promising direction. Another semi-automatic solution, known as “Human-in-the-Loop,” was proposed by Gebauer et al. (2023), which integrates ML with human annotation decisions.

Del Alamo et al. (2022) systematically mapped the landscape of automated analysis of privacy policies. They highlighted a growing interest in such solutions, noting that most of the research has focused on legal compliance that emerged following the introduction of GDPR. While this approach generally yields promising results, they emphasized the need for further research in the field. Automation of a privacy policy-based solution was also proposed to handle a bypass of the OSN’s privacy settings by the act of information sharing by the user, resulting in data leakage (Patsakis et al., 2014). This approach, however, relies on a new mechanism and aims to solve a problem different from the current one. Hosseini et al. (2021) acknowledged the significance of automating privacy policy processes and identified the substantial challenge posed by the diverse structures of such policies. They proposed a preprocessing tool designed to standardize privacy policies into a unified format, facilitating future research endeavors. While their work was automated by applying NLP methodologies, and did not require website cooperation, they mainly focused on preparation of the textual document for research purposes. Woodring et al. (2024) introduced *Privacify*, an application aimed at enhancing the understandability of privacy policies. Their work focuses on simplifying complex text and not on parameterizing the policy. Alshamsan and Chaudhry (2022) underscored the importance of preprocessing in privacy policy classification using ML tools, exemplified by their application of these tools to the OPP-115 corpus (Wilson et al., 2016). This principle is particularly relevant to our work.

Zaeem et al. (2020) introduced the PrivacyCheck, a free online ML based tool, claiming an 80% increase in user consultation of privacy policies. A notable feature of PrivacyCheck is its Competitor Analysis Tool (CAT), which maintains a dataset of privacy policy summaries from various sites within specific markets and recommends superior alternatives to users based on privacy policy quality. However, given the significant user burden and cognitive laziness inherent in this issue, in real-world scenarios, it is unlikely that users will engage in comparison shopping between sites based on their privacy policy quality. Addressing privacy policy violations, Wang et al. (2018) proposed a methodology to ensure consistency between policy implementation and the privacy policy. Our study, however, addresses a different research question, which deals with the problem of ensuring that the implementation is consistent with the privacy policy. Harkous et al. (2018) introduced Poliosis (Automated Framework for Privacy Policy Analysis), which was potentially the first fully automated tool for analyzing privacy policies. However, Poliosis is constrained by its privacy taxonomy, a limitation we overcome with a preset collection of key sentences. Amos et al. (2021) automated the investigation of privacy policies by developing a web crawler to curate past and present data policies, analyzing them by tracking terminology. This research effectively provides a comprehensive review of privacy policy transparency, specifically in disclosing standard tracking technologies and third parties. Its main contribution lies in the extensive statistical analysis enabled by a vast dataset, including 1,071,488 English-language privacy policies.

The main challenges identified in the literature include non-cooperative providers, users’ cognitive laziness, and the dynamic nature of privacy policies. By leveraging AI and ML techniques, this research seeks to reduce cognitive load, improve user comprehension, and support organizational compliance efforts. To this end, by structuring privacy policies in a manner that enhances user understanding and engagement while lowering cognitive burden, the proposed methodology facilitates more informed privacy-related decision-making in sophisticated and evolving digital environments. The methodology relies on a two-layer artificial intelligence process design to addresses these gaps comprehensively. Although prior studies have considered some of the challenges noted above, none has simultaneously addressed all of the critical gaps required to construct an effective and operational solution.

## 3 Structuring privacy policy

### 3.1 Goal definition

This study builds upon principles similar to those used in constructing the P3P protocol, which aimed to automate the structuring of privacy policies. However, unlike the P3P approach, which relied on website cooperation and thus ultimately failed, our approach eliminates this requirement. Our objective is to develop an AI agent capable of structuring privacy policies using resources that are located solely on the users’ side. The AI agent will take the websites’ existing textual privacy policy as input and generate values for predefined parameters, thereby representing the original unstructured textual policy in a structured template.

### 3.2 General approach

To achieve the study objective, a two-layered AI-based process methodology is proposed, which comprises three main phases:

Phase A: A set of textual privacy policies is collected from various websites, constituting the raw data for the process. A fundamental set of parameters is established, and each privacy policy is manually classified based on these parameters.

Phase B: A fundamental set of textual propositions representing the values of the parameters is generated. Subsequently, by employing an NLP technique, these propositions are associated with each policy in the form of probabilities. The outcome of this phase is a labeled dataset containing extracted features that will serve as training data for the machine.

Phase C: Using an ML technique, a machine is trained to classify a textual privacy policy according to its features. The outcome of this phase is a set of rules that can be employed in real-time and in a fully automated process to structure a given privacy policy.

### 3.3 The methodology

**Figure 1:**
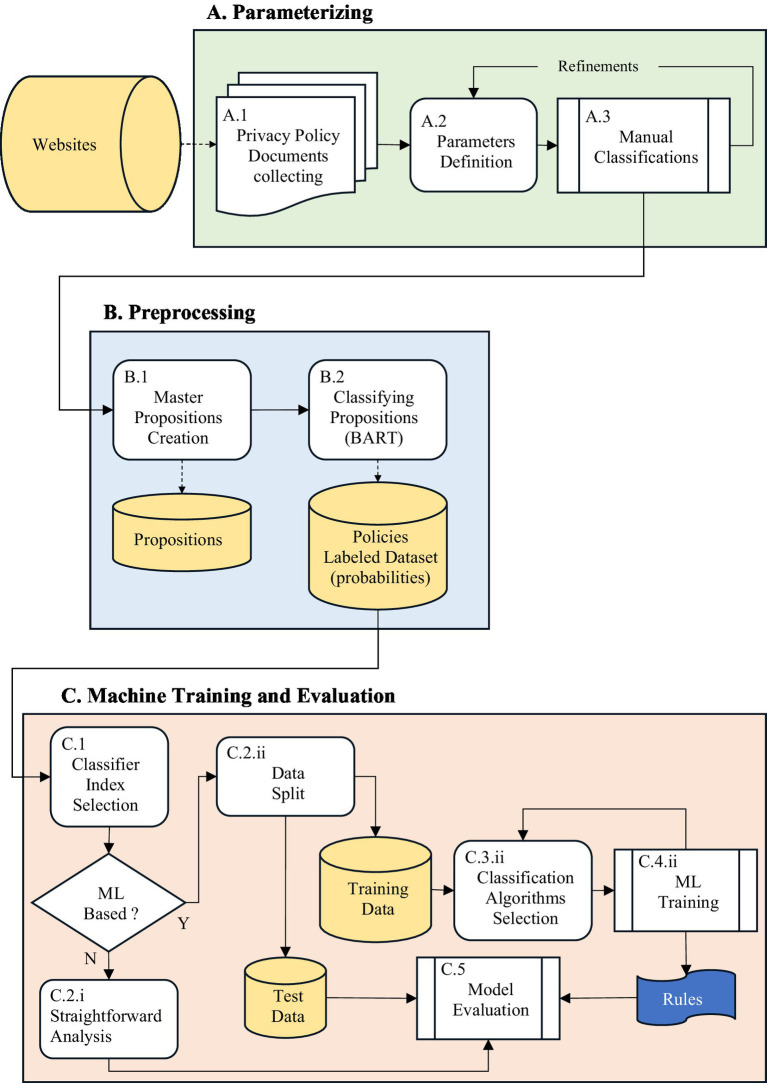
The process of structuring privacy policy is divided into three main phases: **(A)** Parameterizing, **(B)** Preprocessing, **(C)** Machine training and evaluation.

The entire process of structuring a privacy policy is depicted in Figure 1. As depicted, the process is divided into three main phases as described above. In the following description, each process is denoted by an index, corresponding to the figure.

The process starts with phase A, wherein the policy structure parameters are established. This phase is entirely manual. Initially, in step A.1, a set of *n* textual privacy policies is generated from various websites such as Google or AliExpress. This set is denoted $\mathrm{PL}=\{pl_{1},pl_{2},\ldots ,pl_{n}\}$. Subsequently, in step A.2, utilizing the P3P parameters and newly generated parameters resulting from the review of collected policies and existing literature, an initial set of *m* parameters is defined. This set is denoted $\mathrm{PM}=\{pm_{1},pm_{2},\ldots ,pm_{m}\}$, (the set used in the empirical study is described in Table 1). In step A.3, each policy $pl_{i}$ is manually classified according to each parameter $pm_{j}$, i.e., for each privacy policy, we note whether each parameter exists or not, and if it exists, to what extent. As the classification process yields insights, this step may iterate with Step A.2, allowing a refinement of the parameter set PL. For example, the parameter’ Data Collection’ describes the types of data collected by the website. This parameter might have several values such as ‘Personal Data’, ‘Media’, Preferences’, and more. “AliExpress” for example, will receive the values’ Financial’ and ‘Personal Data’. Upon completion of this phase, a set of definitions of parameters and a dataset of privacy policies labeled by these parameters are available.

**Table 1:** The selected parameters and their range of possible values.

Parameter	Explanation	Type	Possible values
First party use	Website data retention policy (Does the website save users’ data for future use?)	Binary	0—No
			1—Yes
Data collection	Types of data collected by the website	Categorial	1—Personal data (e.g., name, address)
			2—Media (e.g., photos, videos, music)
			3—Preferences (e.g., browsing history)
			4—Sound (e.g., audio and recordings)
			5—Finances (e.g., credit cards, purchases)
			6—Geographic identifiers (e.g., location, language)
			7—Infrastructure (e.g., IP address, browser)
			8—Sensitive data (e.g., passwords)
Third party use	The practice regarding the disclosure of user data to third parties. (Does the website reveal (to a third party) users’ data?)	Binary	0—No
			1—Yes
Policy change	The practice for notifying users about changes to the website’s privacy policy. (Does the website notify users about privacy policy changes?)	Binary	0—No
			1—Yes
Data security	The means used by the website to protect the user’s data.	Categorial	1—Two types of data security protocols
			2—General data security limitations
			3—Other protocols
			4—PCI DSS security protocol
User control	Does the user have control over their private data saved on the website (can edit or delete it at any time)?	Binary	0—No
			1—Yes

In Phase B, the objective is to generate essential data for the machine training (ML) and evaluation (scheduled in Phase C). To construct the textual proposition layer for each parameter, we assembled a set of propositions representing the different linguistic formulations through which a parameter may appear in privacy policies. Some propositions were extracted word-for-word (or near-word-for-word) from the collected policies, while others were manually written to capture alternative phrasings, explicit negative constructions, and edge-case scenarios. This mixed construction ensured that the model encountered both naturally occurring expressions and systematically designed linguistic variants, enabling reliable semantic evaluation across heterogeneous policy texts.

For each parameter, the resulting set of propositions is denoted $\mathrm{PP}=\{pp_{₁},\ldots ,pp_{k}\}$ . Each privacy policy was evaluated independently against each proposition using the pretrained BART-large model without fine-tuning. During evaluation, the segmented policy text served as the input sequence and the proposition as the target sequence; no engineered prompting template was applied beyond this default input–output formulation. The model outputs a continuous probability score for each proposition, forming the probability matrix $PL_{\mathrm{PP}}$ of dimension n×k , where n is the number of policies and *k* is the number of propositions for the parameter. Each entry $pl_{\mathrm{PP}}[i,j]$ represents the estimated likelihood that proposition *j* is semantically supported by policy *i*. Because the propositions are evaluated independently, their probabilities do not enforce mutual exclusivity, and positive and negative formulations of the same semantic concept may both receive nonzero values. This independence is intentional because the purpose of Phase B is to collect semantic evidence from multiple linguistic variants rather than enforce categorical interpretation at this stage.

To convert continuous probabilities into parameter-level decisions, thresholds were determined experimentally for each parameter through iterative examination of classification plausibility. When aggregating probabilities across propositions, for instance, computing an average score for positive or negative formulations, complementary propositions were aligned by inversion where necessary. This inversion is applied exclusively for aggregation and does not affect the independent probability assignments produced by the model. This procedure does not impose complementarity in Phase B: contradictory propositions are not required to sum to 1. In contrast, Phase C includes an optional binary evaluation method in which explicitly contradictory propositions are treated as complementary and normalized for the purpose of binary decision-making. This normalization applies only within the controlled binary mechanism of Phase C and is not part of the probabilistic structure of Phase B.

In Phase C.1, one of two main approaches is employed: a straightforward analysis (denoted C.2.i in the figure) and an ML analysis (denoted C.2.ii in the figure). The straightforward analysis is divided into two different calculation methods (which may be considered sub-approaches). The first calculation method attempts to eliminate extreme values by applying the median, calculated as $M_{i}=\text{MEDIAN}(\mathrm{pl}\_pp_{i,\forall j})$ for i∈{1…n},j∈{1…m} , across all propositions for each parameter and each policy. A threshold $T_{i}$ is set for each parameter, through a trial-and-error process. The parameter is considered true if the median is greater than or equal to the threshold, i.e.:

$$\text{Parameter}=\{\begin{array}{cc} \text{TRUE} & M_{i}\geq T_{i}\\ \text{FALSE} & \text{otherwise} \end{array}$$

The second method uses only two propositions (∣PP∣=2 ). These propositions are formulated in a contradictory manner. For instance, proposition 1 states, “We update this privacy policy without prior notice,” while proposition 2 states, “You will be notified of policy changes.” BART assigns a grade between 1 and 0 to each of these contradictory propositions. The sum of these grades equals 1, making them complementary probabilities. In this approach, the parameter is determined as the proposition with a grade greater than 0.5. Both methods are assessed later in step C.5.

In the ML analysis approach, the data is first split into training and test sets in step C.2.ii. Then, some classification algorithms are chosen in step C.3.ii, and the model is trained using these algorithms in step C.4.ii. For each parameter, we applied the following ML algorithms: Decision Tree, Random Forest, Logistic Regression, KNN, SVM, and Neural Network. The best algorithm for each parameter is chosen based on prediction performance. In this ML approach, PL_PP served as the classifier, and the desired predicted data is the information yielded in step A.3. Each model is evaluated in step C.5 by applying it to the test data.

## 4 Empirical study

We applied a set of *n* = 49 privacy policy documents sourced from various websites and platforms (step A.1) to evaluate the methodology. The policies were collected in November 2022 using a purposeful heterogeneity sampling strategy designed to ensure variability across sectors such as e-commerce, social media, financial services, transportation, entertainment, software providers, and regulatory bodies. In the selection process, the popularity of the websites was also considered, aiming to produce a representative dataset by means of usage portion. All policies were publicly available and written in English. The complete list of websites included in the dataset is as follows: Google, AWS, AliExpress, Meta, TikTok, YouTube, Waze, Wix, Bookings.com, WhatsApp, Apple, Wolt, Visa, Mastercard, Airbnb, Uber, Spotify, Samsung, WordPress, Instagram, McDonald’s, FDA, Oracle, Zara, Coca-Cola, Xiaomi, Nasdaq, Walmart, Air Canada, Lufthansa, Shopify, Netflix, Adobe, Starbucks, Shoppers, Decathlon, Walt Disney, American Eagle, Lululemon, SAP, JetBrains, MySQLCode, Cadens, Epic Games, UnitedHealthGroup, Slack, Salesforce, JPMorgan, and Johnson & Johnson.

Each policy was manually labeled according to *m* = 6 parameters derived from existing models and policy reviews (steps A.2 and A.3). Three undergraduate engineering students served as annotators and worked independently following a written guideline specifying definitions, textual indicators, and decision rules. A small shared subset of policies was annotated jointly with the authors to ensure consistent interpretation and refine the guideline. The entire process was conducted under the authors’ supervision. Inter-annotator agreement was not computed because the purpose of this step was to generate sufficiently reliable labels for model training rather than to assess annotator variability. Any inconsistencies identified during the pilot stage were resolved through collaborative discussion. Several parameters permitted multiple simultaneous values (e.g., the Data Collection parameter could include “Personal Data,” “Financial Data,” “Preferences,” and others).

For each parameter, between 2 and 10 textual propositions were constructed, depending on the breadth of the parameter. Some propositions were extracted directly from the policies, while others were manually written to capture alternative linguistic formulations and edge-case phrasing. Examples for manually created propositions include: “*No data sharing occurs with third parties without explicit user consent*” and “*Users cannot request the deletion of their data once it has been processed*.” Examples for directly extracted propositions include: “*Users have the right to request deletion of their information within 30 days*” and “*We may share aggregated, non-identifiable data with trusted service providers*.” These propositions were evaluated independently against each policy using a pretrained BART-large model without fine-tuning. For each parameter, the resulting $PL_{\mathrm{PP}}$ matrix contained the supporting probability for each proposition by each policy. Probabilities across propositions did not sum to 1 because contradictions were intentionally not enforced in Phase B.

To convert continuous proposition probabilities into discrete labels used in Phase C, parameter-specific thresholds were selected (0.65–0.90) based on iterative inspection of plausibility. Thresholding was applied only after probability generation, ensuring consistent and reproducible label construction. Multi-value parameters were encoded using a multi-hot representation so that each possible value corresponded to a separate binary indicator.

For the machine-learning performance evaluation, the dataset was split randomly into a training set and a test set. Policies indexed 0–34 (a total of 35 policies) were used exclusively for training, and policies indexed 35–48 (a total of 14 policies) were used exclusively for testing. Since the policies’ indexes are arbitrary, the selection is practically random. No cross-validation or separate validation set was applied. All classifiers were trained using the default hyperparameters provided by scikit-learn, and no hyperparameter tuning was performed. Binary parameters (e.g., First Party Use, Third Party Use, Policy Change, User Control) were treated as binary classification tasks. Parameters that may assume multiple values simultaneously (e.g., Data Collection, Data Security) were treated as multi-label classification tasks. Multi-label targets were encoded using multi-hot vectors, and models that do not natively support multi-label prediction were trained using scikit-learn’s one-vs-rest mechanism. Given the small dataset (*n* = 49), overfitting is a potential concern. Future work may incorporate k-fold cross-validation or repeated train-test splits to provide more robust performance estimates.

## 5 Results

### 5.1 Preliminary survey

**Figure 2:**
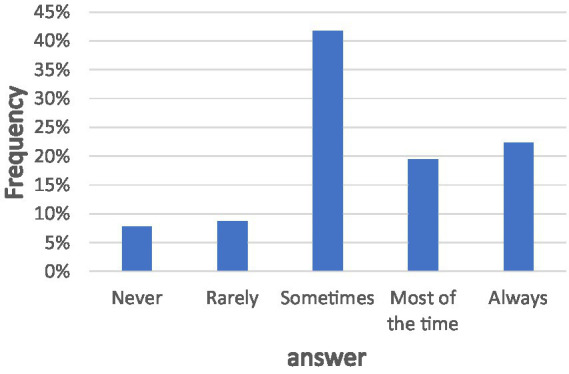
The breakdown of responses to the inquiry concerning the reading of privacy policies. The *x*-axis describes the answer provided, whether the participant bothered to read the privacy policies, and the *y*-axis represents the frequencies of each answer.

As described in the Introduction section, we first conducted a preliminary survey to better understand how users perceive and interact with privacy policy documents. An anonymous sample of *n* = 103 valid participants was asked about their attitudes toward privacy policies and their actual reading behavior. The distribution of responses is depicted in Figure 2. Only 40% of respondents reported that they know what a privacy policy is, and there was no significant correlation between self-reported privacy concern and the approach to the privacy policy document, r(101)=0.183,p=0.064 . These findings indicate that, despite the central role privacy policies are intended to play in protecting users, a substantial portion of users either do not understand them or do not engage with them in practice. This insight reinforces the motivation for developing automated methods that can extract and present the essential content of such documents in a more accessible and structured form.

### 5.2 Parameter distributions

After a thorough examination of the 49 privacy policies in the dataset, we identified the possible range of values for each of the six parameters defined in our framework. While the dataset comprises 49 policies, the selection process ensured diversity across industries and types of websites, providing a representative sample for initial evaluation of the methodology. The parameters and their respective sets of possible values are summarized in Table 1, which specifies, for each parameter, its conceptual meaning, type (binary or categorical), and the corresponding values used in the analysis.

**Figure 3:**
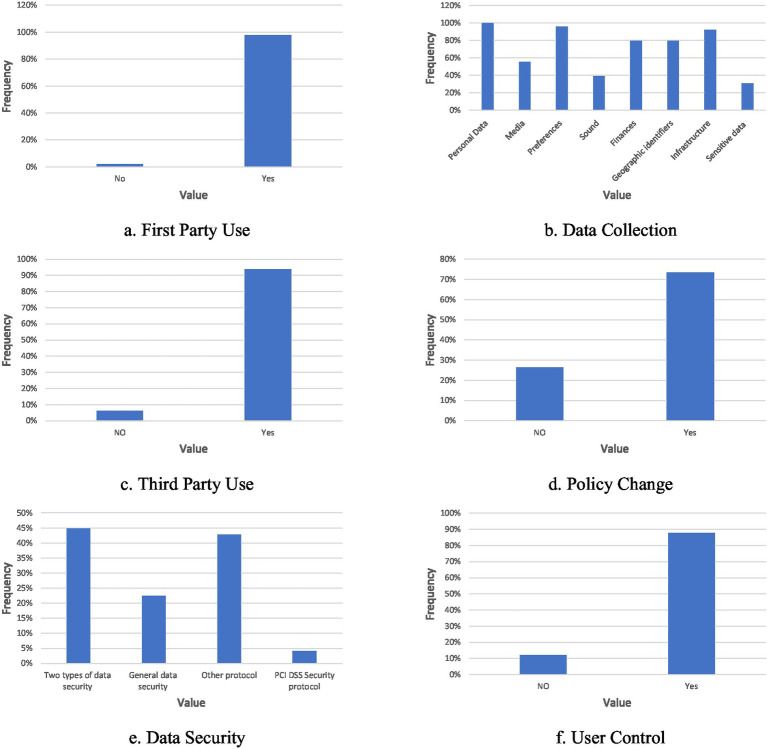
The distributions of the values for each of the parameters: **(a)** First Party Use, **(b)** Data Collection, **(c)** Third Party Use, **(d)** Policy Change, **(f)** Data Security, **(g)** User Control. Each panel represents a single parameter, where the x-axis describes the possible values and the y-axis depicts the frequencies.

For each policy, each parameter was manually evaluated (step A.3), and the resulting distributions of their values are depicted in Figure 3. Each panel in this figure corresponds to one parameter. The *x*-axis shows the possible values of that parameter, and the *y*-axis shows the frequency with which each value appears across all policies. For binary parameters, such as First Party Use, Third Party Use, Policy Change, and User Control—the sum of the frequencies equals 100%, since each policy takes exactly one of the two possible values. In contrast, for non-binary parameters such as Data Collection and Data Security, a single policy may exhibit more than one value simultaneously (for example, a policy may collect both personal data and financial data), so the sum of frequencies can exceed 100%.

The distributions reveal several clear patterns. The majority of policies indicate that the website retains user-collected data (First Party Use). Most policies also state that user data are shared with third parties and that users are permitted to modify their stored data (Third Party Use and User Control). With respect to information types, all websites in the sample retain personal user data, and most retain data related to user preferences, infrastructure, finances, and geographic identifiers. In contrast, fewer than half of the websites retain sensitive data or sound and media data. Regarding policy change notifications, a substantial portion of the sampled websites inform users about changes to the privacy policy, although 26% do not provide such warnings. Finally, with respect to Data Security, only a small number of websites explicitly refer to GDPR or PCI DSS, while most rely on two-factor authentication or other, less prevalent specified protocols. These parametric value-space and distributions, summarized in Table 1 and Figure 3, provide the empirical backdrop for evaluating the performance of the automated analysis methods.

**Table 2:** F1 score obtained from each analysis for each of the tested parameters.

Analysis	Parameter	First party use	Data collection	Third party use	Policy change	Data security	User control
Straightforward	Median	0.97	0.72	0.77	0.75	0.61	0.93
	Contradicting propositions	0.98	0.63	0.86	0.73	0.27	0.06
Machine Learning (ML)		1	0.76	0.87	0.85	0.68	0.87

### 5.3 Performance of straightforward and machine-learning (ML) approaches

To assess the effectiveness of the proposed methodology, we compared two families of analysis approaches applied to the $PL_{\mathrm{PP}}$ matrices: straightforward aggregation methods and ML models. The straightforward methods and the machine-learning method were evaluated on the same manually labeled dataset, and their F1 scores for each parameter are reported in Table 2.

The straightforward analyses included a median-based approach and an approach based on contradictory propositions. In the median approach, the parameter value is inferred by aggregating proposition-level probabilities with the median operator. As shown in Table 2, this method achieved high F1 scores for several parameters, including First Party Use and User Control, indicating that the central tendency of the probabilities provides a reliable signal when the relevant textual cues are clear and consistently expressed across policies. The contradictory-proposition approach, which relies on explicit pairs of opposing propositions, performed well for some parameters, particularly Third Party Use, where the underlying linguistic formulations are relatively direct. However, for parameters such as Data Security and User Control, this method yielded substantially lower F1 scores, reflecting the difficulty of representing complex or heterogeneous practices solely through pairs of complementary statements.

**Figure 4:**
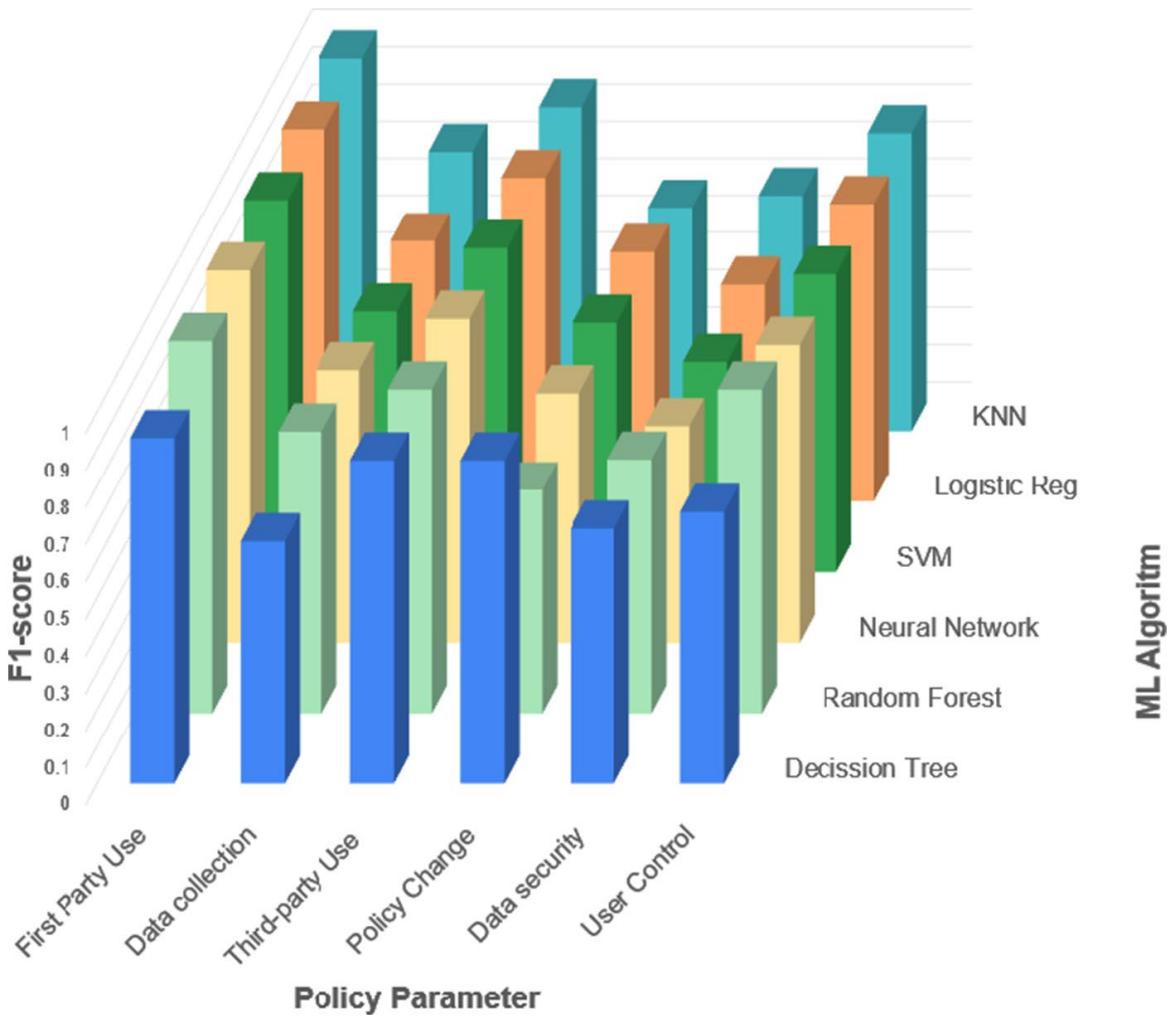
The overall performance of each machine learning algorithm for each parameter.

The ML approach treated the problem as a supervised classification task, using the labels derived from the thresholded probabilities as targets. As shown in Table 2, the ML method outperformed both straightforward methods for almost all parameters; the only exception is the median-based method for the User Control parameter, which achieved a comparable F1 score. The overall performance profiles of the different ML algorithms for each parameter are presented in Figure 4, where the *x*-axis denotes the policy parameter, the *y*-axis denotes the algorithm, and the *z*-axis represents the F1 score.

Based on these results, we selected, for each parameter, the algorithm that achieved the highest performance. Random Forest was chosen for First Party Use, Data Collection, and User Control, while Decision Tree was selected for Third Party Use, Policy Change, and Data Security. The detailed performance indices of the chosen algorithms, including accuracy, precision, recall, and F1 score, are reported in Table 3. The prediction process yielded excellent results for First Party Use, Data Collection, Policy Change, and User Control, with F1 scores between 0.84 and 1.00. For Third Party Transfer, the results were moderate, with an F1 score of 0.80. The only parameter for which the results were unsatisfactory was Data Security, with an F1 score of 0.50, reflecting the substantial variability and ambiguity observed in how security practices are described in the policies.

**Table 3:** Machine-learning algorithm performance for each policy parameter.

Parameter	Accuracy	Precision	Recall	F1 Score
First party use	1.00	1.00	1.00	1.00
Data collection	0.77	0.79	0.89	0.84
Third party use	0.87	0.75	0.87	0.80
Policy change	0.87	0.89	0.87	0.86
Data security	0.73	0.54	0.47	0.50
User control	0.87	0.89	0.87	0.84

**Figure 5:**
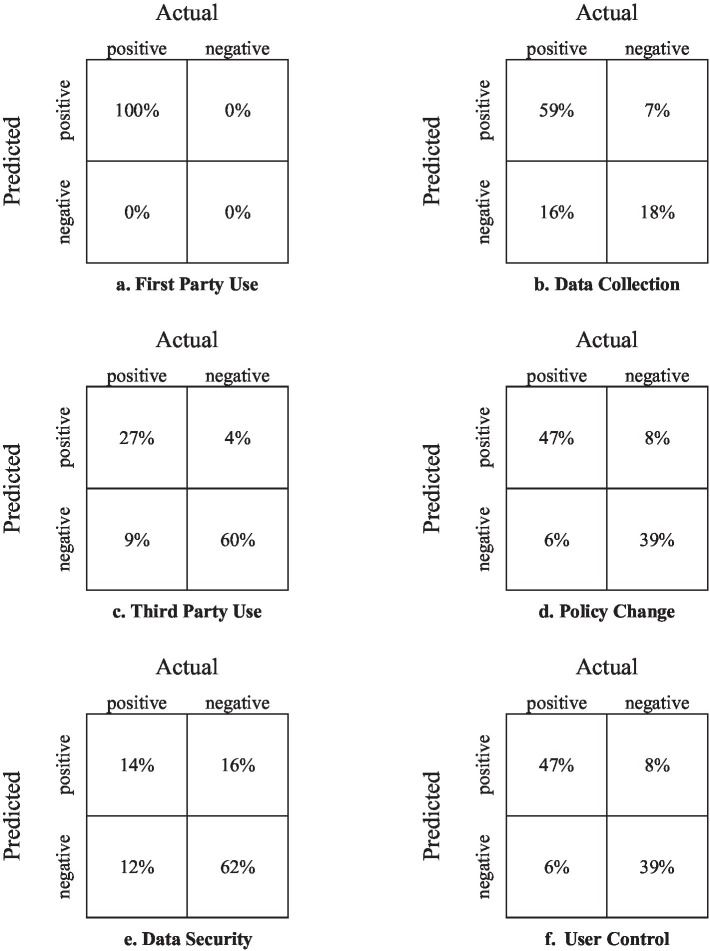
Confusion matrices for each of the parameters: **(a)** First Party Use, **(b)** Data Collection, **(c)** Third Party Use, **(d)** Policy Change, **(e)** Data Security, **(f)** User Control.

A more detailed report of the ML results is depicted in Figure 5. For each of the six parameters, the confusion metrics are provided when the values are indicated in percentage (of the total amount of the policies that were included). Again, it can be observed that, besides Data Security, the process yields high-accuracy results for all other parameters. A closer inspection of the Data Security reference in the privacy policies reveals that the text addressing this parameter is more diverse. This artifact may identify the reason for the relatively low accuracy in deciphering the Data Security parameter, and it calls for further research. For this end, several options may be adopted, e.g., fine-tuning of the related propositions or expanding the sample size used to train the machine. It is worth mentioning that the values of the errors that appear in the confusion matrices are semantic beyond statistical indices. The false-positive values (upper-right cell of each matrix) indicated that a privacy threat was wrongly identified. This may mislead users into not using the site, resulting in a loss of benefits. On the other hand, the false-negative values (lower-left cell of each matrix) indicated that a privacy threat was not identified. This may mislead users into using the site, potentially causing a privacy violation. Notably, besides one parameter, these values are low and most satisfying.

Together, Tables 2, 3 and Figures 4, 5 show that the proposition-based representation, when combined with supervised ML models, can successfully reconstruct key structural properties of privacy policies from unstructured text.

**Table 4:** An example of the scores (probabilities) of each proposition that belongs to the third party use parameter when evaluating Google’s privacy policy.

Proposition (simplified)	Probability (Google)
“we share personal information”	0.9889
“the data is being transferred to third party”	0.9632
“we will share your personal data with third parties”	0.9464
“third parties have access to your personal data”	0.9233
“Guest user personal data may be shared with third parties”	0.9835

### 5.4 Working example

To demonstrate how the proposed methodology operates in practice, we present a concrete example using the Google privacy policy from the empirical dataset. This example illustrates: (a) how the textual propositions interact with the original text; (b) how the $PL_{\mathrm{PP}}$ probability matrix is generated; and (c) how the system’s output compares to the ground-truth labels assigned by human annotators.

The following section from Google’s privacy policy (Phase A input), served as the textual basis for evaluating the Third Party Use parameter: “We do not share your personal information with companies, organizations, or individuals outside of Google except in the following cases: with your consent… for external processing… for legal reasons…” This text contains explicit conditional statements describing circumstances in which personal data is shared with third parties. Accordingly, the human annotators (*n* = 3) assigned the value “Yes” for the Third Party Use parameter in the ground-truth dataset. For this parameter, the system evaluated Google’s policy against a set of predefined textual propositions. The BART model produced probability scores for each proposition, as described in Table 4. All the scores were above the experimentally determined threshold of 0.9.

These values, taken directly from the $PL_{\mathrm{PP}}$ matrix generated in Phase B, indicate that the model consistently detected semantic support for third-party data transfer statements.

Using the parameter-specific threshold for the Third Party Use classification, the aggregated proposition probabilities surpassed the cutoff value, leading the system to predict the label “Yes.” This prediction aligned with the human-assigned ground-truth label, indicating that the model correctly classified the policy. Accordingly, in this case, the model successfully reproduced the parameter value determined by the human annotators. This example demonstrates the entire reasoning chain in the methodology: (a) identification of relevant text in the policy; (b) proposition-level semantic evaluation; (c) probability aggregation and thresholding; and (d) direct comparison between model output and manually assigned labels. It also demonstrates how the system handles real-world conditional statements, which are common in privacy policies and can often be difficult for users to interpret manually.

## 6 Discussion

This study addresses the longstanding challenge of making privacy policies accessible and interpretable for both human users and automated systems. Privacy policies are designed to safeguard individual privacy and have been widely examined in the literature (Mhaidli et al., 2023). Nevertheless, their practical effectiveness remains limited, if exists at all, due to their complexity, frequent revisions, cognitive demands on readers, and the perception that the reading effort exceeds the benefit gained. Although the full text of such policies is legally binding and necessary for formal compliance, in everyday contexts, many of their essential elements can be distilled into a set of clear parameters, such as whether a website shares user data with third parties. This gap between regulatory requirements and users’ needs motivates the current work.

A privacy policy contains crucial operational information that enables downstream analyses, for example, when examining conflicts in service collaboration (Wu and Liu, 2014). Yet users often encounter these documents as lengthy, dense text rather than as structured statements of data practices. In some cases, clarity may even benefit service providers, e.g., more salient presentation of privacy terms has been shown to influence consumers’ willingness to pay a premium in e-commerce environments (Tsai et al., 2011). The methodology presented in this work aims to bridge these gaps by automatically transforming the unstructured textual document into a structured list of parameter values. Prior research has demonstrated the feasibility of semi-automated extraction of data practices (Wilson et al., 2018), and the current study extends this foundation by introducing a fully automated methodology based on a two-layer AI framework. The empirical evaluation yielded strong performance for five of the six parameters examined, achieving F1 scores between 0.8 and 1.0, thereby demonstrating the viability of automated, text-based structuring of privacy policies without requiring cooperation from website owners.

The lack of standardization across privacy policy formats and the dynamic nature of these documents further complicate user comprehension and adherence, particularly when websites must update their policies frequently to reflect changes in the site privacy behavior, and to comply with evolving regulations (Wagner, 2023). By reducing a policy through automated extraction to a predefined set of parameters, each indexed with a value, the proposed methodology mitigates these challenges. The strength of the approach lies in its autonomy—the agent requires only the textual privacy policy, which is itself a legally binding document. Unlike earlier frameworks such as P3P, which failed due to low adoption by website operators (Schwartz, 2009; EPIC, 2000), the present methodology does not depend on any external markup, cooperation, or standard compliance.

At the same time, the increasing availability of personal data and the growing role of AI in decision-making processes introduce risks of privacy invasion (Lepri et al., 2021). The methodology developed here can extend beyond consumer privacy policies to domains such as evaluating compliance with open-data policies in scientific publishing, thereby supporting reproducibility (Hardwicke et al., 2018). An enhanced AI agent built upon this methodology could also assist users in understanding the implications of sharing information across digital platforms, aligning with regulatory intentions such as the GDPR’s emphasis on transparency and user-centric data management.

An additional advantage of the methodology is that it can be deployed locally, avoiding reliance on cloud-based processing. This stands in contrast to tools such as Grammarly, which analyze user documents on remote servers and whose privacy policy, according to Klusaitė (2024), permits sharing personal data with companies and governmental agencies if required by law. While both Grammarly and the current agent involve access to user-related information, local execution ensures that no sensitive data leaves the user’s device, thereby significantly reducing privacy risks. Training the model requires substantial computational resources, but inference requires very little, making the agent suitable for deployment even on lightweight devices.

The methodology also opens several avenues for future development. Internally, improved proposition sets could enhance performance, particularly for parameters with substantial linguistic variability, such as Data Security. One possible direction involves generating and refining propositions using additional AI layers, applied offline and iteratively. Expanding the training dataset could also improve generalizability, though it necessitates additional manual labeling. Externally, the methodology can be embedded within user-facing systems. A dedicated interface could allow users to specify their privacy preferences, enabling the agent to compare these preferences with structured outputs extracted from any website. This design resembles earlier tools such as AT&T Privacy Bird, which aimed to provide real-time notices based on P3P labels (w3.org, 2020), but differs in that the present solution does not require any cooperation or markup from service providers (which is the primary cause for the failure of the P3P project). The system could automatically notify or block actions when privacy-policy values conflict with the user’s stated preferences, similar to the behavior of antivirus software.

At a broader level, AI-based support for alleviating the cognitive burden placed on users is increasingly essential in digital environments where both legitimate and harmful automated processes shape user experience (Di Pietro and Cresci, 2021). However, AI systems may also assist providers in generating privacy policies, thereby transforming structured content into unstructured text, as demonstrated in the automated generation of physician letters (Hou et al., 2025). These dual uses underscore the need for tools that help users interpret machine-generated documents as well.

The methodology also has limitations. Although the evaluation metrics treat classification errors symmetrically, users may not. For example, mistakenly warning that personal data *is* collected may deter beneficial use of a service, whereas mistakenly signaling that personal data is *not* collected may expose users to privacy risks. Future systems could allow users to adjust the balance between these two errors according to their personal preferences and the sensitivity (or cost) associated with each error type. Additionally, while the methodology demonstrated strong performance on current policies, future privacy policies may adopt new formats, including multimedia content or relocated critical information (e.g., to terms-of-service sections), which may reduce model performance. Furthermore, even perfectly interpreting the privacy policy text does not guarantee alignment with real-world practices, as shown in studies of policy inconsistency in virtual personal assistant ecosystems (Shafei et al., 2024). These concerns fall outside the present study’s scope but highlight directions for further investigation. The methodology may also extend to internal organizational documents such as Privacy Impact Assessments (PIAs), which guide compliance processes (Iwaya et al., 2024).

Finally, the emergence of privacy-policy designs intentionally resistant to machine interpretation represents a possible adversarial trend. Analogous to CAPTCHA tests designed to differentiate humans from machines (Moradi and Keyvanpour, 2015), websites could craft policies that satisfy regulatory requirements while inhibiting automated analysis. Addressing such strategies would require ongoing maintenance and adaptation of the agent. Nevertheless, the methodology holds promise in two complementary architectures: as a standalone assistant enabling users to understand the essential meaning of privacy policies and as a modular component in broader decision-support frameworks that incorporate user intentions and information-sharing contexts. For example, automated tools for assessing information-sharing risk (Guarino et al., 2022) currently overlook platform privacy practices; integrating the output of the proposed agent could significantly enhance such systems.

## Data Availability

The datasets presented in this study can be found in online repositories. The names of the repository/repositories and accession number(s) can be found at: https://github.com/YuvalBar246/Privacy-Policy-Final-Project.

## References

[ref1] AG (2023). What is a privacy policy? Available online at: https://www.oaic.gov.au/privacy/your-privacy-rights/your-personal-information/what-is-a-privacy-policy (Accessed June 2025).

[ref2] Alibaba Group (2022). AliExpress.com Privacy Policy. Available online at: https://terms.alicdn.com/legal-agreement/terms/suit_bu1_aliexpress/suit_bu1_aliexpress201909171350_82407.html (Accessed June 2025).

[ref3] Alić M. (2023). “Privacy notice informativeness: in a search for benchmark” in 2023 46th MIPRO ICT and Electronics Convention (MIPRO) (New York: IEEE), 1496–1500.

[ref4] Alshamsan A. R. Chaudhry S. A. (2022). Machine learning algorithms for privacy policy classification: a comparative study. New York: IEEE, 214–219.

[ref5] Amos R. Gunes A. Eli L. Mihir K. Arvind N. Jonathan M. . (2021). “Privacy policies over time: curation and analysis of a million-document dataset” in Proceedings of the web conference 2021, Ljubljana Slovenia: ACM. 2165–2176.

[ref6] Anderson A. H. (2006). “A comparison of two privacy policy languages: EPAL and XACML” in Proceedings of the 3rd ACM workshop on Secure web services, New York, NY, United States: ACM. 53–60.

[ref7] Beatty P. Reay I. Dick S. Miller J. (2007). P3P adoption on e-commerce web sites: a survey and analysis. IEEE Internet Comput. 11, 65–71. doi: 10.1109/MIC.2007.45

[ref8] Bélanger F. James T. L. (2020). A theory of multilevel information privacy management for the digital era. Inf. Syst. Res. 31, 510–536. doi: 10.1287/isre.2019.0900, 19642375

[ref9] Blanco C. García-Saiz D. Rosado D. G. Santos-Olmo A. Peral J. Maté A. . (2022). Security policies by design in NoSQL document databases. J. Inf. Secur. Appl. 65:103120. doi: 10.1016/j.jisa.2022.103120

[ref10] California Law (2003). Chapter 22. Internet privacy requirements. Available online at: https://leginfo.legislature.ca.gov/faces/codes_displayText.xhtml?division=8.&chapter=22.&lawCode=BPC (Accessed June 2025).

[ref11] Chenthara S. Ahmed K. Wang H. Whittaker F. (2019). Security and privacy-preserving challenges of e-health solutions in cloud computing. IEEE Access 7, 74361–74382. doi: 10.1109/ACCESS.2019.2919982

[ref12] Costante E. Sun Y. Petković M. Den Hartog J. (2012). “A machine learning solution to assess privacy policy completeness: (short paper)” in Proceedings of the 2012 ACM workshop on privacy in the electronic society, 91–96.

[ref13] Cranor L. F. (2003). P3P: making privacy policies more useful. IEEE Secur. Privacy 1, 50–55. doi: 10.1109/MSECP.2003.1253568

[ref14] Custers B. van der Hof S. Schermer B. (2014). Privacy expectations of social media users: the role of informed consent in privacy policies. Policy Internet 6, 268–295. doi: 10.1002/1944-2866.POI366

[ref15] Del Alamo J. M. Guaman D. S. García B. Diez A. (2022). A systematic mapping study on automated analysis of privacy policies. Computing 104, 2053–2076. doi: 10.1007/s00607-022-01076-3

[ref16] Di Pietro R. Cresci S. (2021). Metaverse: security and privacy issues. New York: IEEE, 281–288.

[ref17] Dorraji S. E. Barcys M. (2014). Privacy in digital age: dead or alive?! Regarding the new EU data protection regulations. Soc. Technol. 4, 306–317. doi: 10.13165/ST-14-4-2-05, 41459400

[ref18] Draper N. A. (2017). From privacy pragmatist to privacy resigned: challenging narratives of rational choice in digital privacy debates. Policy Internet 9, 232–251. doi: 10.1002/poi3.142

[ref19] Earp J. B. Anton A. I. Aiman-Smith L. Stufflebeam W. H. (2005). Examining internet privacy policies within the context of user privacy values. IEEE Trans. Eng. Manag. 52, 227–237. doi: 10.1109/TEM.2005.844927

[ref20] Elliott D. Soifer E. (2022). AI technologies, privacy, and security. Front. Artif. Intell. 5:826737. doi: 10.3389/frai.2022.826737, 35493613 PMC9044077

[ref21] EPIC (2000). Pretty poor privacy: an assessment of P3P and internet privacy. Available online at: https://archive.epic.org/reports/prettypoorprivacy.html (Accessed June 2025).

[ref22] Feth D. (2017). “Transparency through contextual privacy statements” in Mensch und Computer 2017 - Workshopband: Spielend einfach interagieren, Books on Demand, Hamburg, 290.

[ref23] Gebauer M. Mashhur F. Leschke N. Grünewald E. Pallas F. (2023). A human-in-the-loop approach for information extraction from privacy policies under data scarcity. arXiv preprint arXiv:2305.15006. doi: 10.48550/arXiv.2305.15006

[ref24] Ghazinour K. Albalawi T. (2016). A usability study on the privacy policy visualization model. New York: IEEE, 578–585.

[ref25] Google (2023). Google privacy & terms. Available online at: https://policies.google.com/privacy?hl=en-IL&fg=1 (Accessed March 2025).

[ref26] Gopal R. D. Hidaji H. Kutlu S. N. Patterson R. A. Yaraghi N. (2023). Law, economics, privacy: implications of government policies on website and third-party information sharing. Inf. Syst. Res. 34, 1375–1397. doi: 10.1287/isre.2022.1178, 19642375

[ref27] Guarino A. Malandrino D. Zaccagnino R. (2022). An automatic mechanism to provide privacy awareness and control over unwittingly dissemination of online private information. Comput. Netw. 202:108614. doi: 10.1016/j.comnet.2021.108614

[ref28] Hardwicke T. E. Mathur M. B. MacDonald K. Nilsonne G. Banks G. C. Kidwell M. C. . (2018). Data availability, reusability, and analytic reproducibility: evaluating the impact of a mandatory open data policy at the journal cognition. R. Soc. Open Sci. 5:180448. doi: 10.1098/rsos.180448, 30225032 PMC6124055

[ref29] Harkous H. Fawaz K. Lebret R. Schaub F. Shin K. G. Aberer K. . (2018). Polisis: Automated analysis and presentation of privacy policies using deep learning. 27th USENIX Security Symposium (USENIX Security 18), 531–548.

[ref30] Hinds J. Williams E. J. Joinson A. N. (2020). “It wouldn't happen to me”: privacy concerns and perspectives following the Cambridge analytica scandal. Int. J. Hum. Comput. Stud. 143:102498. doi: 10.1016/j.ijhcs.2020.102498

[ref31] Hoang N. P. Niaki A. A. Gill P. Polychronakis M. (2021). Domain name encryption is not enough: Privacy leakage via IP-based website fingerprinting. Cornell University, arXiv:2102.08332. doi: 10.48550/arXiv.2102.08332

[ref32] Hosseini H. Degeling M. Utz C. Hupperich T. (2021). Unifying privacy policy detection. Proc. Priv. Enhanc. Technol. 2021, 480–499. doi: 10.2478/popets-2021-0081, 40909103

[ref33] Hosseini S. Seilani H. (2025). The role of agentic AI in shaping a smart future: a systematic review. Array 26:100399. doi: 10.1016/j.array.2025.100399

[ref34] Hou Y. Bert C. Gomaa A. Lahmer G. Höfler D. Weissmann T. . (2025). Fine-tuning a local LLaMA-3 large language model for automated privacy-preserving physician letter generation in radiation oncology. Front. Artif. Intell. 7:1493716. doi: 10.3389/frai.2024.1493716, 39877751 PMC11772293

[ref35] Huang W. Liu B. Tang H. (2019). Privacy protection for recommendation system: a survey. J. Phys. Conf. Ser. 1325:12087. doi: 10.1088/1742-6596/1325/1/012087

[ref36] Ingber A. S. Su C. C. (2024). Protecting children of the TikTok era: a discourse analysis in the absence of law. Policy Internet. Hoboken, New Jersey, USA. 17. doi: 10.1002/poi3.431

[ref37] Iwaya L. H. Alaqra A. S. Hansen M. Fischer-Hübner S. (2024). Privacy impact assessments in the wild: a scoping review. Array 23:100356. doi: 10.1016/j.array.2024.100356

[ref38] Jensen C. Potts C. (2004). “Privacy policies as decision-making tools: an evaluation of online privacy notices” in Proceedings of the SIGCHI conference on human factors in computing systems, Vienna Austria: ACM. 471–478.

[ref39] Jha N. Trevisan M. Vassio L. Mellia M. (2022). The internet with privacy policies: measuring the web upon consent. ACM Trans. Web 16, 1–24. doi: 10.1145/3555352,

[ref40] Jin H. Luo Y. Li P. Mathew J. (2019). A review of secure and privacy-preserving medical data sharing. IEEE Access 7, 61656–61669. doi: 10.1109/ACCESS.2019.2916503,

[ref41] Kanakia H. Shenoy G. Shah J. (2019). Cambridge analytica--a case study. Indian J. Sci. Technol. 12, 1–5. doi: 10.17485/ijst/2019/v12i29/146977

[ref42] Karegar F. Pettersson J. S. Fischer-Hübnerr S. (2020). The dilemma of user engagement in privacy notices: effects of interaction modes and habituation on user attention. ACM Trans. Privacy Secur. 23, 1–38. doi: 10.1145/3372296

[ref43] Klusaitė L. (2024). Does Grammarly share your sensitive data with third parties? Available online at: https://nordvpn.com/blog/is-grammarly-safe/ (Accessed July 2025).

[ref44] Krumay B. Klar J. (2020). “Readability of privacy policies” in IFIP annual conference on data and applications security and privacy (Cham: Springer), 388–399.

[ref45] Kumaraguru P. Cranor L. Lobo J. Calo S. (2007). “A survey of privacy policy languages” in Workshop on usable IT security management (USM 07): proceedings of the 3rd symposium on usable privacy and security (New York: ACM).

[ref46] Lawrence N. D. Montgomery J. (2024). Accelerating AI for science: open data science for science. R. Soc. Open Sci. 11:231130. doi: 10.1098/rsos.231130, 39169971 PMC11336680

[ref47] Lepri B. Oliver N. Pentland A. (2021). Ethical machines: the human-centric use of artificial intelligence. IScience 24:102249. doi: 10.1016/j.isci.2021.102249, 33763636 PMC7973859

[ref49] Li H. Yu L. He W. (2019). The impact of GDPR on global technology development. J. Glob. Inf. Technol. Manag. 22, 1–6. doi: 10.1080/1097198X.2019.1569186

[ref50] Linden T. Khandelwal R. Harkous H. Fawaz K. (2019). The privacy policy landscape after the GDPR. Cornell University, arXiv preprint arXiv:1809.08396. doi: 10.48550/arXiv.1809.08396

[ref51] Liu Y. Li N. (2018). Retrieving hidden friends: a collusion privacy attack against online friend search engine. IEEE Trans. Inf. Forensics Secur. 14, 833–847. doi: 10.1109/TIFS.2018.2866309,

[ref52] Lutz C. Hoffmann C. P. Bucher E. Fieseler C. (2018). The role of privacy concerns in the sharing economy. Inf. Commun. Soc. 21, 1472–1492. doi: 10.1080/1369118X.2017.1339726

[ref53] Mc Laughlin G. H. (1969). SMOG grading-a new readability formula. J. Read. 12, 639–646.

[ref54] McDonald A. M. Cranor L. F. (2008). The cost of reading privacy policies. Isjlp 4:543.

[ref55] Meta (2023). Available online at: https://www.facebook.com/privacy/policy/?entry_point=comet_dropdown (Accessed March 2025).

[ref56] Mhaidli A. Selin F. An D. Gina H. Mukund S. Lee M. . (2023). “Researchers’ experiences in analyzing privacy policies: challenges and opportunities” in Proceedings on privacy enhancing technologies, Lausanne, Switzerland: PoPETs. 287–305.

[ref57] Mokrosinska D. (2018). Privacy and autonomy: on some misconceptions concerning the political dimensions of privacy. Law Philos. 37, 117–143. doi: 10.1007/s10982-017-9307-3

[ref58] Moradi M. Keyvanpour M. (2015). CAPTCHA and its alternatives: a review. Secur. Commun. Netw. 8, 2135–2156. doi: 10.1002/sec.1157

[ref59] Mouloua S. A. Ferraro J. Mouloua M. Matthews G. Copeland R. R. (2019). Trend analysis of cyber security research published in HFES proceedings from 1980 to 2018. Los Angeles, CA: SAGE Publications, 1600–1604.

[ref60] Muneer A. Razzaq S. Farooq Z. (2018). Data privacy issues and possible solutions in E-commerce. J. Account. Market. 7:1000294. doi: 10.4172/2168-9601.1000294

[ref61] NHS 75 (2023). What is a privacy notice? Available online at: https://www.england.nhs.uk/contact-us/privacy-notice/find-out-what-a-privacy-notice-is/ (Accessed June 2025).

[ref62] Nippert-Eng C. E. (2019). Islands of privacy. Chicago: University of Chicago Press.

[ref63] Patsakis C. A. Z. A. Papageorgiou A. Galván-López E. (2014). Distributing privacy policies over multimedia content across multiple online social networks. Comput. Netw. 75, 531–543. doi: 10.1016/j.comnet.2014.08.023

[ref64] Paul P. Aithal P. (2019). “Mobile applications security: an overview and current trend” in Proceedings of national conference on research in higher education, learning and administration, 112–121.

[ref65] Puaschunder J. (2021). “A utility theory of privacy and information sharing” in Encyclopedia of information science and technology. 5th ed (Hershey: IGI Global), 428–448.

[ref66] Qing H. Ibrahim R. Nies H. W. (2024). Comprehensive location privacy enhanced model. iScience 27:111412. doi: 10.1016/j.isci.2024.111412, 39687010 PMC11647162

[ref67] Regan P. M. (2002). Privacy as a common good in the digital world. Inf. Commun. Soc. 5, 382–405. doi: 10.1080/13691180210159328

[ref68] Rhahla M. Allegue S. Abdellatif T. (2021). Guidelines for GDPR compliance in big data systems. J. Inf. Secur. Appl. 61:102896. doi: 10.1016/j.jisa.2021.102896

[ref69] Robillard J. M. Feng T. L. Sporn A. B. Lai J. A. Lo C. Ta M. . (2019). Availability, readability, and content of privacy policies and terms of agreements of mental health apps. Internet Interv. 17:100243. doi: 10.1016/j.invent.2019.100243, 30949436 PMC6430038

[ref70] Sadeh N. . (2013). The usable privacy policy project. Pittsburgh: Carnegie Mellon University.

[ref71] Schwartz A. (2009). Looking back at P3P: lessons for the future. Washington, DC: Center for Democracy & Technology.

[ref72] Shafei H. A. Gao H. Tan C. C. (2024). Measuring privacy policy compliance in the Alexa ecosystem: in-depth analysis. Comput. Secur. 144:103963. doi: 10.1016/j.cose.2024.103963

[ref73] Sharma V. You I. Andersson K. Palmieri F. Rehmani M. H. Lim J. (2020). Security, privacy and trust for smart mobile-internet of things (M-IoT): a survey. IEEE Access 8, 167123–167163. doi: 10.1109/ACCESS.2020.3022661

[ref74] Shvartzshnaider Y. Apthorpe N. Feamster N. Nissenbaum H. (2019). “Going against the (appropriate) flow: a contextual integrity approach to privacy policy analysis” in Proceedings of the AAAI conference on human computation and crowdsourcing, Washington State: PKP Publishing Services, Skamania Lodge. 162–170.

[ref75] Smith W. P. Tabak F. (2009). Monitoring employee e-mails: is there any room for privacy? Acad. Manag. Perspect. 23, 33–48. doi: 10.5465/AMP.2009.45590139

[ref76] Srinath M. Wilson S. Giles C. L. (2020). Privacy at scale: introducing the privaseer corpus of web privacy policies. Cornell University, arXiv preprint arXiv:2004.11131. doi: 10.48550/arXiv.2004.11131

[ref77] Steinfeld N. (2016). “I agree to the terms and conditions”:(how) do users read privacy policies online? An eye-tracking experiment. Comput. Hum. Behav. 55, 992–1000. doi: 10.1016/j.chb.2015.09.038

[ref78] Tsai J. Y. Egelman S. Cranor L. Acquisti A. (2011). The effect of online privacy information on purchasing behavior: an experimental study. Inf. Syst. Res. 22, 254–268. doi: 10.1287/isre.1090.0260, 19642375

[ref79] USAgov (2023). USA.gov privacy and security policies. Available online at: https://www.usa.gov/privacy-security (Accessed June 2025).

[ref80] Van Schaik P. . (2018). Security and privacy in online social networking: risk perceptions and precautionary behaviour. Comput. Hum. Behav. 78, 283–297. doi: 10.1016/j.chb.2017.10.007

[ref81] w3.org (2020). Privacy bird user study. Available online at: https://www.w3.org/2002/p3p-ws/pp/privacybird.pdf (Accessed March 2025).

[ref82] W3C (2018). The platform for privacy preferences 1.0 (P3P1.0) Specification. Available online at: https://www.w3.org/TR/P3P/ (Accessed March 2025).

[ref83] Wagner I. (2023). Privacy policies across the ages: content of privacy policies 1996--2021. ACM Trans. Privacy Secur. 26, 1–32. doi: 10.1145/3590152

[ref84] Wang X. Xue Q. Bokaei H. M. Rocky S. Travis D. B. Jianwei N. . (2018). “GUILeak: tracing privacy policy claims on user input data for android applications” in Proceedings of the 40th international conference on software engineering, Gothenburg Sweden: ACM. 37–47.

[ref85] Wilson S. Schaub F. Liu F. Sathyendra K. M. Smullen D. Zimmeck S. . (2018). Analyzing privacy policies at scale: from crowdsourcing to automated annotations. ACM Trans. Web 13, 1–29. doi: 10.1145/3230665,

[ref86] Wilson S. Florian S. Abhilash D. A. Frederick L. Sushain C. Giovanni L. P. . (2016). “The creation and analysis of a website privacy policy corpus” in Proceedings of the 54th annual meeting of the association for computational linguistics (Volume 1: Long Papers), Berlin, Germany: Association for Computational Linguistics. 1330–1340.

[ref87] Winkler S. Zeadally S. (2016). Privacy policy analysis of popular web platforms. IEEE Technol. Soc. Mag. 35, 75–85. doi: 10.1109/MTS.2016.2554419

[ref88] Woodring J. Perez K. Ali-Gombe A. (2024). Enhancing privacy policy comprehension through Privacify: A user-centric approach using advanced language models. Comput. Secur. 145:103997. doi: 10.1016/j.cose.2024.103997

[ref89] Wu Z. Liu Y. (2014). Knowledge augmented policy conflict analysis for services collaboration. Knowl.-Based Syst. 62, 11–27. doi: 10.1016/j.knosys.2014.02.019

[ref90] Wu Z. Shen S. Zhou H. Li H. Lu C. Zou D. (2021). An effective approach for the protection of user commodity viewing privacy in e-commerce website. Knowl.-Based Syst. 220:106952. doi: 10.1016/j.knosys.2021.106952

[ref91] Yang J. Yessenov K. Solar-Lezama A. (2012). A language for automatically enforcing privacy policies. ACM SIGPLAN Not. 47, 85–96. doi: 10.1145/2103621.2103669

[ref92] Zaeem R. N. Safa A. Alex I. Jake N. Isabelle R. Vinay S. . (2020). Privacycheck’s machine learning to digest privacy policies: competitor analysis and usage patterns. New York: IEEE, 291–298.

[ref93] Zakhary S. Benslimane A. (2018). On location-privacy in opportunistic mobile networks, a survey. J. Netw. Comput. Appl. 103, 157–170. doi: 10.1016/j.jnca.2017.10.022

